# Serum level of vitamin D in patients with recurrent aphthous stomatitis: A systematic review and meta‐analysis of case control studies

**DOI:** 10.1002/cre2.794

**Published:** 2023-10-03

**Authors:** Roya Safari‐Faramani, Mohsen Salehi, Saman Ghambari Haji Shore, Neda Omidpanah

**Affiliations:** ^1^ Research Center for Environmental Determinants of Health, School of Public Health Kermanshah University of Medical Sciences Kermanshah Iran; ^2^ Students Research Committee Kermanshah University of Medical Sciences Kermanshah Iran; ^3^ Department of Oral and Maxillofacial Medicine, School of Dentistry Kermanshah University of Medical Sciences Kermanshah Iran

**Keywords:** recurrent aphthous stomatitis, vitamin D

## Abstract

**Objectives:**

Recurrent aphthous stomatitis (RAS) is an ulcerative condition with unknown etiology. The effect of vitamin D in the etiology of RAS is still a matter of controversy. In this study, we aimed at review the available evidence on the role of vitamin D deficiency in RAS etiology.

**Material and Methods:**

PubMed, Cochrane Library for Systematic Reviews, ISI Web of Science, Scopus, and EmBase were systematically searched for evidence on RAS and vitamin D up to January 2020. Retrieved records were screened and assessed by two of the authors independently. Newcastle−Ottawa scale was used to assess the quality of individual studies. AMSTAR tool was used for assessing the quality of the study.

**Results:**

Eight studies including 383 healthy control and 352 patients with RAS were eligible for the meta‐analysis. Serum vitamin D levels were significantly lower in RAS patients. The weighted mean difference was −7.90 (95% CI: −11.96 to −3.85).

**Conclusions:**

The results highlighted the importance of vitamin D deficiency in the etiology of RAS. However, more studies are needed to reach a robust decision. The observed association between vitamin D and RAS is probably due to the effect of vitamin D on the immune system.

## 1 BACKGROUND

Recurrent aphthous stomatitis (RAS) is the most common lesion of the oral mucosa, with a prevalence of from 5% to 66% in the general population (Akintoye & Greenberg, 2005; Scully, 2006). RAS is diagnosed by ruling out other possible causes of stomatitis (Krisdapong et al., 2012; Tabolli et al., 2009). RAS is morphologically divided into three categories: minor, major, and herpetiform, the most common form of which is minor and accounts for 85% of RAS lesions and it does not leave scars. The major type is more widespread and its size is larger than 10 mm, and its recovery is accompanied by the creation of scars. The herpetic type is a form of multiple deep wounds with irregular edges (Feng et al., 2015).

Although the underlying causes of RAS are unclear, some research has shown that systemic factors, including genetics, immunological, and hematological abnormalities, as well as other factors such as trauma, smoking, stress, nutritional deficiencies, and allergies, are among the potential causes (Rivera‐Hidalgo et al., 2004; Slebioda et al., 2014). These initiating factors cause the secretion of preinflammatory cytokines by infiltration of leukocytes against a specific area of the oral mucosa depending on the extent and severity of the disease (Rivera‐Hidalgo et al., 2004; Slebioda et al., 2014; Tabolli et al., 2009).

Hydroxyvitamin D (25‐OHD), also known as vitamin D, is a type of fat‐soluble vitamin. It is produced in the skin through exposure to sunlight and can also be obtained from certain foods and supplements. Vitamin D deficiency can occur due to insufficient sunlight exposure, reduced availability, or certain medications such as glucocorticoids, antiretroviral drugs, or anticonvulsants (Straube et al., 2015). Vitamin D receptors (VDR) are present in many tissues throughout the body, and a lack of 25‐OHD has been associated with various health conditions, including musculoskeletal disorders, metabolic and autoimmune diseases, respiratory and cardiovascular issues, cancer, psychiatric disorders, chronic pain, and hypothyroidism (Adorini, 2005; Taheriniya et al., 2021).

Recently, the role of vitamin D in the etiopathogenesis of RAS has been highlighted, (Ali, 2019; Bahramian et al., 2018).

1,25 Dihydroxyvitamin D, a bioactive form of vitamin D3, a steroid hormone, has a crucial role in calcium and bone metabolism (Adorini, 2005). Vitamin D endocrine system has the property of regulating immune and inflammatory responses. The cells of the innate and adaptive immune system, such as macrophages, T cell, B cell, express the dendritic cells that receive vitamin D (VDR) and are involved in the production and response to vitamin D. The net effect of vitamin D on the immune system is to increase intrinsic immunity with multiple adaptive immune systems (Adorini et al., 2004).

The role of vitamin D in the development of RAS has been a subject of debate in recent studies. Several investigations have examined the potential connection between low levels of vitamin D and the occurrence of RAS, yielding conflicting findings. While certain studies have indicated a correlation between vitamin D deficiency and the onset of RAS, (Ali, 2019; Bahramian et al., 2018; Khabbazi et al., 2015; Öztekin & Öztekin, 2018) others have not definitively established the role of vitamin D deficiency in RAS lesions (Krawiecka et al., 2017; Suhail et al., 2019). The objective of the current study was to conduct a systematic review of the available evidence concerning the influence of vitamin D deficiency on the pathogenesis of RAS.

## 2 METHODS

### 2.1 Protocol and registration

The Preferred Reporting Items for Systematic Review and Meta‐Analyses (PRISMA) guideline followed to report the results of this systematic review (Liberati, 2009) and was registered on the International prospective register of systematic reviews, PROSPERO (CRD42019138725).

### 2.2 Focused question (based on PICO criteria)

The study was designed to answer if the level of serum vitamin D is different between patients with RAS and healthy controls. The study question was clarified based on the PICO categories (i.e., population, intervention [exposure in this study], comparison group, and outcomes).

### 2.3 Search strategy

A systematic search was conducted in electronic databases (Scopus, Web of Science, PubMed, Embase, and Cochrane Library) up to January 2020, without language restrictions. The search terms used “Aphthous” or “Oral ulcer” or “Sutton's disease” or “recurrent aphthous were Stomatitis” and “25‐hydroxy vitamin D” or “vitamin D.”

### 2.4 Eligibility criteria and study selection process

Inclusion criteria were observational studies that reported levels vitamin D in saliva or serum, between the RAS patients and healthy controls. Exclusion criteria included commentaries, letters to the editor, editorials, case reports, reviews/systematic reviews, conference abstracts, book chapters, and studies with irrelevant data.

Based on the inclusion criteria, the eligibility of each article was evaluated by reviewing their titles and abstracts. Two reviewers independently evaluated the titles and abstracts for this purpose. Disagreements were resolved following discussion with a third reviewer. Next, the full‐texts of the suggested potential eligible articles were retrieved and screened. The reasons for exclusion of articles were documented.

### 2.5 Data extraction

Two reviewers independently extracted the data from the full‐texts of articles based on a predetermined form and a third reviewer checked the extracted data. The following details were extracted: authors, publication year, sample size in each group, mean age of the participants, male percent, laboratory method, and serum or saliva level of vitamin D in each group.

### 2.6 Risk of bias assessment

Newcastle−Ottawa quality assessment scale for case control studies was used to assess the risk of bias in the individual studies (Wells et al., 2014).

The Newcastle−Ottawa scale is based on three main domains including selection, comparability, and exposure. This tool also served to classify the quality of each study as either high (7 or above), fair (between 4 and 7), or low (less than 4). One of the authors performed this quality assessment. The quality assessment was conducted by one author. To assess the quality of the present systematic review, AMSTAR 2 (Assessing the Methodological Quality of Systematic Reviews) tool was used (Shea et al., 2017).

### 2.7 Statistical methods

The standard mean difference and its 95% confidence interval (CI) were calculated for each study using Review Manager 5.3 (RevMan 5.3; Cochrane Collaboration). These values were used to assess the difference in salivary and serum vitamin D levels between patients with RAS and healthy controls. A *p*‐value less than .05 was considered statistically significant. To evaluate the heterogeneity among the studies, the *I* ^2^ statistic was used. A *p*‐value less than .1 (*I* ^2^ > 50%) indicated significant heterogeneity, leading to the use of a random‐effects model for the analysis.

Funnel plot analysis was performed using the Comprehensive Meta‐Analysis version 2.0 (CMA 2.0) software. Both Egger's and Begg's tests were used to assess publication bias, with a *p*‐value less than .05 (two‐tailed) indicating significant publication bias.

The unit of measurement for salivary and serum vitamin D levels in this meta‐analysis was ng/mL.

The study protocol was funded and registered in the Research Committee of Kermanshah University of Medical Sciences.

## 3 RESULTS

### 3.1 Study selection

**Figure 1:**
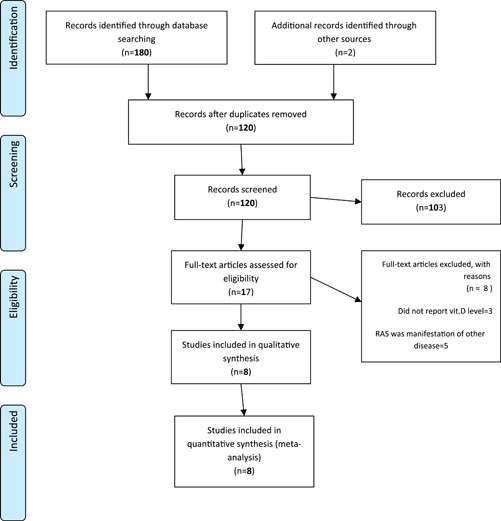
Flow diagram of literature search.

After removing the duplicates literature search yielded 120 records. After excluding the irrelevant title and abstracts, 17 studies were chosen to be potentially eligible for the review. Of which, three did not report vitamin D levels; one did not have a control group and in the other five, RAS was the manifestation of the other disease. Finally, eight studies were included for the final review. Details of the study selection are depicted in Figure 1.

### 3.2 Study characteristics

The characteristics of the included case‐control studies are presented in Table 1.

**Table 1:** Characteristics of studies included in the systematic review on comparing the level of vitamin D levels in RAS patients and the healthy controls.

Author	Year of publication, country	Mean age, male percent (%)		Method	RAS type	Sample size		Mean ± SD of serum vitamin D (ng/mL)	
		RAS patients	Healthy controls			RAS patients	Healthy controls	RAS patients	Healthy controls
Funda Tamer	2018, Turkey	34−25	33.9−30	NR	Minor	20	20	13.6 ± 6.5	20.9 ± 10
Al‐Amad Suhail	2019, United Arab Emirates	34−66	31−66	ECLIA	Idiopathic minor	52	52	53.6 ± 24.6	51.1 ± 26.9
Ayla Bahramian	2018, Iran	38.8−61.5	40.8−65.4	ECLIA	Idiopathic minor	26	26	33.07 ± 12.41	50.89 ± 9.3
Alireza Khabbazi	2014, Iran	33.4**−**60.8	34.1−61.2	ELISA	Idiopathic minor	46	49	12.1 ± 7.7	27.4 ± 9.7
Ewa Krawiecka1	2017, Poland	34.15−36.4	32.05−24.2	ECLIA	Minor, major, herpetiform	66	66	16.81 ± 8.45	19.22 ± 10.44
Aynure Öztekin	2018, Turkey	31.2−37.5	27.4−45.7	ECLIA	Idiopathic minor	40	70	11 ± 7.03	16.4 ± 10.19
Nalbantoğlu, A.	2019, Turkey	8.7−46.1	7.6−51.4	ELISA	Idiopathic minor	72	70	16.4 ± 8.6	23.1 ± 11.5
Ali, N. S. M.	2019, Iraq	36.4−0	33.64−0	ELISA	Idiopathic minor	30	30	13.9 ± 12.72	22.08 ± 17.779

Abbreviations: ELISA, enzyme‐linked immunosorbent assay; RAS, recurrent aphthous stomatitis.

Eight studies including 383 healthy control and 352 patients with RAS were eligible for the meta‐analysis. The mean age of the participants ranged from 8.7 to 36.4 in RAS patients and 7.6 to 40.8 in healthy controls.

Out of eight studies included in the meta‐analysis, two studies were from Iran (Bahramian et al., 2018; Khabbazi et al., 2015), three from Turkey (Nalbantoğlu & Nalbantoğlu, 2019; Öztekin & Öztekin, 2018; Tamer & Avcı, 2019), one from Poland (Krawiecka et al., 2017), one from United Arab Emirates (Suhail et al., 2019), and one from Iraq (Ali, 2019).

One study (Bahramian et al., 2018) reported vitamin D levels on saliva and serum and the rest of studies on serum. Five studies assessed the association between serum levels of vitamin D and RAS variables such as duration, severity, and frequency (Ali, 2019; Khabbazi et al., 2015; Krawiecka et al., 2017; Liberati, 2009; Suhail et al., 2019).

The measurement method of serum levels of vitamin d in three (Ali, 2019; Khabbazi et al., 2015; Nalbantoğlu & Nalbantoğlu, 2019) studies was ELISA (enzyme‐linked immunosorbent assay) and four (Bahramian et al., 2018; Krawiecka et al., 2017; Öztekin & Öztekin, 2018; Suhail et al., 2019) studies was ECLAI (electro‐chemiluminescencbinding assay) and one study did not report (Tamer & Avcı, 2019).

Mean age, male percent, rest of the data are shown in Table 1.

### 3.3 Risk of bias assessment

Results of the risk of bias assessment for individual studies are presented in Table 2. The total score for all the included studies was more than 5.

**Table 2:** Risk of bias assessment of primary studies based on Newcastle−Ottawa scale for case control studies.

Author	Selection	Comparability	Exposure	Total score
Funda Tamer	2	1	2	5
Al‐Amad Suhail	3	1	2	6
Ayla Bahramian	3	1	2	6
Alireza Khabbazi	3	1	2	6
Ewa Krawiecka1	3	1	2	6
Aynure Öztekin	3	1	2	6
Nalbantoğlu, A.	3	1	3	7
Ali, N. S. M.	2	1	2	5

### 3.4 Meta‐analysis reports

**Figure 2:**
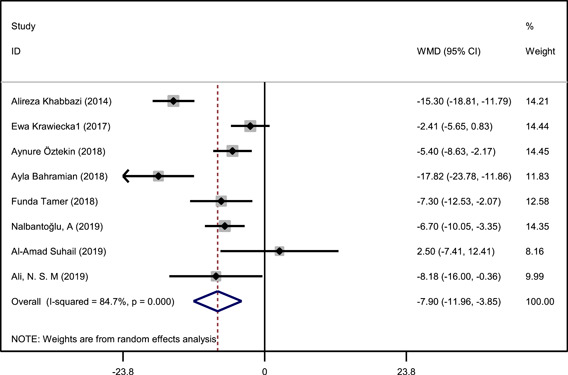
Weighted mean difference (WMD) of vitamin D levels between RAS patients and the healthy controls. Random effects model was applied. RAS, recurrent aphthous stomatitis.

Figure 2 shows serum levels of vitamin D in RAS and healthy controls. Out of the eight included studies, two studies didn't find significant differences between serum vitamin D levels in patients with RAS and healthy controls (Bahramian et al., 2018; Krawiecka et al., 2017). One study demonstrated a significant association between serum levels of vitamin D and number ulcer in patients with RAS (Bahramian et al., 2018).

In general, serum vitamin D levels were significantly lower in RAS patients. The weighted mean difference was −7.90 (95% CI: −11.96 to −3.85) which was significantly differ from zero, (*z* = 3.82, *p* < .0001). Heterogeneity *χ* ^2^ was 45.7 (degree of freedom = 7, *p* < .0001) (Figure 2).

### 3.5 Publication bias

The symmetrical funnel plot using the Begg's and Egger's tests showed low risk of publication bias (the *p* values for the Begg' and Egger's tests were .536 and .851, respectively).

### 3.6 Sensitivity analysis

Sensitivity analysis was performed by omitting one study in turn, with the pooled weighted mean difference varied between −9.04 (95% CI: −10.72 to −7.37) and −5.97 (95% CI: −7.62 to −4.34), supporting the stability of the results.

### 3.7 The quality of the systematic review

The quality of evidence was evaluated using the second version of AMSTAR tool. A score of 15 out of 16 was obtained.

## 4 DISCUSSION

In the present study published evidence on the role of vitamin D deficiency in the occurrence of RAS has been systematically reviewed and the results have been summed up via meta‐analysis. Based on the results, the serum level of vitamin D was significantly lower in patients suffering RAS compared to the healthy controls. However, we did not find an association between a vitamin D deficiency and frequency of ulcers in RAS patients. Previous research has often associated multivitamin deficiency in RAS patients with hematinic deficiencies such as vitamin B12, folic acid, and iron (Chen et al., 2015). However, our systematic review indicates that vitamin D levels appear to be lower in RAS patients compared to controls.

RAS is a painful condition that negatively impacts eating, speech, and oral hygiene. The frequent and severe relapses can significantly reduce the quality of life. The exact cause of RAS is not completely understood (Lopez‐Jornet et al., 2014; Saikaly et al., 2018). The results of the current study consistent with many previous studies that demonstrated Low levels of vitamin D in autoimmune diseases such as rheumatoid arthritis, systemic lupus erythematosus, MS, type 1 diabetes, inflammatory bowel disease, and psoriasis (Bergler‐Czop & Brzezińska‐Wcisło, 2016; Cutolo et al., 2006; Hyppönen et al., 2001; Teichmann et al., 1999).

How vitamin D affects RAS is not yet well understood. Given that the immunological context for RAS has been determined. The VDR and the vitamin D activating enzyme (1‐α‐hydroxylase) are expressed in various immune cells found, including T cells, macrophages, and dendritic cells and in the epithelial tissue of the oral mucosa. Low levels of vitamin D in RAS patients were associated with the VDR, proinflammatory cytokines, strong immunomodulatory effects, and T helper cells. T helper cells induce proinflammatory cytokines, leading to epithelial damage or ulcers (Adorini, 2002; Chen et al., 2015).

Vitamin D deficiency in RAS‐related diseases such as Behcet's syndrome and PFAPA (priodic fever, aphthous stomatitis, pharangits, and cervical adenitis) that one of the main symptoms of which is RAS, has been proven (Aslan et al., 2017; Faydhi et al., 2022). According to Ainure Oztekin study, patients with RAS could benefit from taking vitamin D as a supportive treatment (Öztekin & Öztekin, 2018). Therefore, reducing vitamin D levels might play a role in the development of RAS, for the reasons mentioned above.

Limitations: (1) Studies did not have a similar method for measuring vitamin D levels. (2) Patients with different types of RAS were included in the studies. (3) varying male‐to‐female ratios. (4) The geographical location of the studies, which were seven conducted in Asia. These factors contributed to high heterogeneity between the studies.

Strengths: (1) we had no publication bias across studies. (2) One study being of high quality and seven studies being of moderate quality according to the NOS tool. (3) Most studies were matched by sex and age. (4) Sensitivity analysis supported the stability of the results.

## 5 CONCLUSION

There was a relationship between low vitamin D in serum and RAS. Therefore, it may be worthwhile to check vitamin D levels in patients with RAS.

## AUTHOR CONTRIBUTIONS

Roya Safari‐Faramani, Mohsen Salehi, and Saman Ghambari Haji Shore conceived and designed the study, formulated the research question, helped in the design of the study, including the screening of the retrieved records, interpretation of the results as well as the draft of this paper. Neda Omidpanah, Mohsen Salehi, and Saman Ghambari Haji Shore screened the retrieved records, extracted the data for this study, and performed the statistical analysis and interpretation of the results as well as the draft of this paper. All three authors have given final approval for this study to be published.

## CONFLICT OF INTEREST STATEMENT

The authors declare no conflict of interest.

## ETHICS STATEMENT

This article does not contain any studies with human participants or animals performed by any of the authors.

## Data Availability

The data will be avaible upon the request.
